# Compositional data analysis of 24-hour movement behaviors and mental health in workers

**DOI:** 10.1016/j.pmedr.2020.101213

**Published:** 2020-09-29

**Authors:** Naruki Kitano, Yuko Kai, Takashi Jindo, Kenji Tsunoda, Takashi Arao

**Affiliations:** a Physical Fitness Research Institute, Meiji Yasuda Life Foundation of Health and Welfare, 150 Tobuki, Hachioji, Tokyo 192-0001, Japan; b Faculty of Social Welfare, Yamaguchi Prefectural University, 3-2-1 Sakurabatake, Yamaguchi, Yamaguchi 753-8502, Japan

**Keywords:** Time-use epidemiology, Sleep, Physical activity, Sedentary behavior, Accelerometer, Work engagement

## Abstract

This cross-sectional study investigated the relationship between 24-hour movement behaviors and mental health among Japanese workers, accounting for the co-dependence of time spent in different behaviors during a single day. This research is part of a prospective cohort study called the Meiji Yasuda LifeStyle study. Participants were 1095 workers in Tokyo, Japan, who underwent annual health check-ups between 2017 and 2018. Time use during workdays and non-workdays was separately evaluated. Behaviors included sleep (self-reported duration), sedentary behavior (SB), light-intensity physical activity (LPA), and moderate- to vigorous-intensity physical activity (assessed with a tri-axial accelerometer). We used the six-item Kessler Psychological Distress scale to evaluate psychological distress and the Utrecht Work Engagement Scale to assess work engagement as indicators for negative and positive mental health, respectively. We performed compositional logistic regression and compositional isotemporal substitution to examine associations between 24-hour movement behaviors and mental health. After controlling for potential confounders, time spent in workdays was significantly associated with higher psychological distress and lower work engagement (*p* < 0.05), respectively. Time spent sleeping was positively correlated with both mental health indicators, whereas time spent in SB or LPA was negatively correlated (*p* < 0.05). During workdays, higher psychological distress and lower work engagement were predicted to be 11.4–26.6% lower when 60 min/day of time spent sleeping were reallocated to SB or LPA. Reallocating time spent on SB or LPA to sleep during workdays may minimize psychological distress and optimize work engagement. Thus, proper time management focused on encouraging sleep may help improve workers’ mental health.

## Highlights

• Time in sleep was favorably associated with mental distress and work engagement.
• Sedentary behavior (SB) was negatively correlated with distress and work engagement.
• Light-intensity physical activity (LPA) was negatively correlated with distress.
• Reallocating time in SB or LPA to sleep was associated with better mental health.

## 1 Introduction

Psychological distress is increasingly prevalent, and approximately 300 million people suffer from depression and anxiety disorders globally (World Health Organization, 2017). Working adults are a major target population for mental wellbeing interventions because workers are routinely exposed to risk factors for psychological distress (e.g., long work hours, job strain, bullying) (Theorell et al., 2015). Much focus has been placed on developing strategies that mitigate negative mental health in workers. However, the management of workers’ mental health should consider both negative and positive psychological states. The latter is associated with better physical health, life satisfaction, and job performance (Shimazu et al., 2015). Particularly, a positive psychological state worth examining is work engagement, defined as feeling positive, and fulfilled when working.

Several epidemiological studies using conventional analytical methods have examined the relationship between workers’ daily movement behaviors and psychological states. Systematic reviews suggest that sleep (Litwiller et al., 2017) and physical activity (PA) (Mammen and Faulkner, 2013, Nishi et al., 2017) have protective effects on workers’ psychological states, whereas the effects of sedentary behavior (SB) are detrimental (Mullane et al., 2017, Munir et al., 2015, Zhai et al., 2015). One criticism of these findings, however, is that daily movement behaviors are co-dependent; only 24 h are available for allocating to sleep, SB, and PA (Chastin et al., 2015, Pedisic, 2014). Therefore, increased time spent on one movement behavior (e.g., sleep) must be compensated by an accompanying decrease in other movement behaviors (e.g., SB, PA, or both). Although this temporal displacement would necessarily influence the health outcomes associated with these behaviors, most research does not adequately adjust for this interrelationship of time use due to collinearity problems (Chastin et al., 2015, Pedisic, 2014).

Compositional data analysis (CoDA) is a statistical approach that can address this criticism, accounting for this co-dependency, and is thus the recommended statistical method for examining the associations between time use and health outcomes (Chastin et al., 2015, Dumuid et al., 2018, Pedisic, 2014). To date, only a few studies are available using CoDA to examine relationships between time use and mental health. Their results have been inconsistent. One study reported a non-significant association between 24-hour movement behaviors and psychological distress (Curtis et al., 2020), whereas two others found that more sleep, SB, and moderate- to vigorous-intensity physical activity (MVPA) were associated with depressive symptoms in adults (del Pozo Cruz et al., 2020) and with overall mental health in the elderly (McGregor et al., 2018). However, no study has used CoDA to investigate these relationships in workers specifically. Such research should account for the known phenomenon of workers using non-workdays to compensate for insufficient sleep (Im et al., 2017) and inactivity (Gubelmann et al., 2017) during workdays. Previous research has proposed the Framework for Viable Integrative Research in Time-Use Epidemiology (Pedisic et al., 2017). The framework suggests that we need to re-examine the relationship between time use and health outcomes, adequately considering the compositional nature of daily time data. It also highlights the need to identify unhealthy patterns of time use among specific population subgroups. In the context, it is expected that our study will help to develop population strategies that promote optimal time-use patterns for workers’ mental health.

Therefore, this cross-sectional study used CoDA to examine how 24-hour movement behaviors during workdays and non-workdays are associated with psychological distress and work engagement.

## 2 Materials and methods

### 2.1 Study design and participants

This research is part of a prospective cohort study called the Meiji Yasuda LifeStyle (MYLS) study, which uses data from annual health check-ups conducted at the Meiji Yasuda Shinjuku Medical Center in Tokyo, Japan (Tsunoda et al., 2014). Participants in this cohort were mainly employees and their spouses living in the capital region. This analysis employed cross-sectional data collected between 2017 and 2018 from 1647 employees in several departments of a life insurance company. Individuals were included if they were assessed with accelerometry, worked during weekdays, and had weekends off. All participants consented to their inclusion in this study. The Ethical Committee of Meiji Yasuda Life Foundation of Health and Welfare approved all protocols (approval no. 28006).

### 2.2 Measurements

#### 2.2.1 Movement behaviors

A tri-axial accelerometer (Active style Pro HJA750-C, Omron Healthcare, Kyoto, Japan) was used to assess movement behaviors while awake. The accelerometer and a health checkup kit were mailed to each participant at least two weeks before the scheduled checkup. Participants were instructed to wear the accelerometer on their hip during waking hours for at least ten days, including both workdays and non-workdays, except during conditions that could potentially damage the device (e.g., water-based activities or contact sports).

This accelerometer has been validated (Ohkawara et al., 2011, Oshima et al., 2010), and its measurement accuracy is comparable to devices widely used by researchers in Western countries (Kurita et al., 2017, Murakami et al., 2016). An epoch-length was set at 60-seconds and estimated metabolic equivalents (METs) were obtained using developer-provided software. Non-wear time was defined in intervals of 20 consecutive minutes with activity counts under the detection limit (Peeters et al., 2013) and valid days were defined as days when participants wore the device for ≥ ten hours (Tudor-Locke et al., 2012). Non-wearing time was subtracted from 24 h to obtain wearing time. Participants were included in the final analysis if they collected data for two or more valid workdays and one or more valid non-workday (Masse et al., 2005). Each 60-second epoch was classified as SB (≤1.5 METs), light-intensity PA (LPA; 1.6–2.9 METs), and MVPA (≥3.0 METs) (Haskell et al., 2007, Pate et al., 2008). Minutes spent in each of these behaviors were aggregated per day and averaged over all valid days.

Average sleep duration was evaluated using the question: “In a typical workday/non-workday, how many hours do you usually sleep, excluding napping?” Sleep duration was expressed as a proportion of 24 h. The remaining hours were then allocated across SB, LPA, and MVPA in proportion to the total time recorded for each behavior (del Pozo Cruz et al., 2020, McGregor et al., 2019).

#### 2.2.2 Mental health

Psychological distress was assessed as an indicator of negative mental health using the Japanese version of the six-item Kessler Psychological Distress Scale (K6) (Furukawa et al., 2008), with confirmed reliability and validity. Data were dichotomized into low and high psychological distress, with a score of five points being the cut-off (Nishi et al., 2018). As an indicator of positive mental health, work engagement was evaluated using a shortened Japanese version of the Utrecht Work Engagement Scale, consisting of three subscales (vigor, dedication, and absorption) (Shimazu et al., 2012). The scale has high internal reliability and good test-retest reliability (Shimazu et al., 2012). This study used three items from the vigor subscale to assess work engagement. The vigor subscale reflects overall work engagement, because each of its three items was strongly correlated with each other (Schaufeli et al., 2006). Data were dichotomized into low (<3 points) or high (≥3 points) work engagement, based on the median of all participants (equivalent to the average score of Japanese employees in epidemiological studies) (Shimazu et al., 2010).

#### 2.2.3 Covariates

Potential confounders based on *a priori* clinical judgment and previous knowledge (Lund et al., 2018) were selected and included as covariates. These variables included age, sex, body mass index (BMI), years of education, self-rated economic status, marital status, health behaviors (alcohol consumption and smoking), job-type, hiring status, and average weekly overtime working hours. Data were collected with a self-administered questionnaire, except for BMI, which was calculated from participant heights and weights (measured using a stadiometer and a medical scale, respectively).

### 2.3 Statistical analysis

R packages *compositions* version 1.40.3 (van den Boogaart and Tolosana-Delgado, 2008) and *robCompositions* version 2.1.0 (Templ et al., 2011) were used for CoDA. All procedures followed previous research on applying CoDA to movement behaviors (Chastin et al., 2015). Analyses were conducted separately for workdays and non-workdays.

Compositional multiple logistic regression (Amagasa et al., 2019) was conducted to examine associations between movement behaviors and mental health. Odds ratios (ORs) and 95% confidence intervals (CIs) were calculated. Before inclusion in the regression model, time-use composition (Sleep, SB, LPA, and MVPA) was expressed as a set of isometric log ratio (ilr) coordinates using a strategy known as pivot coordinate representation. A set of three ilr-coordinates was obtained from the composition so that the first coordinate expressed the relative time spent in one behavior compared to the other three.

(1) $$\begin{array}{c} \mathrm{il}r_{1}=\sqrt{\frac{3}{4}}ln\frac{\mathrm{Sleep}}{{\left(\mathrm{SB}\cdot LPA\cdot MVPA\right)}^{\frac{1}{3}}} \end{array}$$

(2) $$\begin{array}{c} \mathrm{il}r_{2}=\sqrt{\frac{2}{3}}ln\frac{\mathrm{SB}}{{\left(\mathrm{LPA}\cdot MVPA\right)}^{\frac{1}{2}}} \end{array}$$

(3) $$\begin{array}{c} \mathrm{il}r_{3}=\sqrt{\frac{1}{2}}ln\frac{\mathrm{LPA}}{\mathrm{MVPA}} \end{array}$$

In the above ilr-coordinates, for example, ilr_1_ represents the ratio of sleep time to SB, LPA, and MVPA time. We obtained four ilr coordinate systems by iterating the transformations so that each of the four behaviors was a numerator of the first ilr-coordinate. In the regression model, psychological distress and work engagement were dependent variables, while movement behaviors (expressed as ilrs) were independent variables. Model 1 included only these variables. Model 2 was additionally adjusted for age (continuous), sex (male/female), BMI (continuous), education years (continuous), self-rated economic status (very poor or poor/good or very good), marital status (married/unmarried), smoking status (never or former/current smoker), alcohol consumption (never/sometimes/every day), job-type (sales worker/other), hiring status (full-time/other), and overtime working hours (not applicable/<10 h/week/≥10 h/week). The significance of associations for the whole time-use composition and its specific parts was assessed using the likelihood ratio test (LRT) and Wald test. Results for the first ilr coordinate for sleep, SB, LPA, and MVPA were shown, given that the focus is on time spent per behavior relative to the remaining three behaviors.

The results of compositional logistic regression (i.e., OR) are difficult to understand directly/alone and cannot be interpreted as “the change in the odds ratio for a 1 unit (min/day) increase in a behaviour” as in traditional regression analysis. Therefore, to obtain more meaningful information, we conducted a detailed analysis. After confirming the significant associations of time spent in each movement behavior, compositional isotemporal substitution (Amagasa et al., 2019, Dumuid et al., 2019, McGregor et al., 2019) was conducted based on Model 2. In this analysis, the expected percent change in poor mental health was calculated based on pairwise reallocations. Specifically, a fixed duration was reallocated from one movement behavior to another, while keeping the remaining behaviors constant. Estimates were calculated in 10-minute increments up to 60-minutes. Compositional data analysis and the interpretation of the results are described in detail in previous studies (Amagasa et al., 2019, Dumuid et al., 2019, McGregor et al., 2019).

All statistical analyses and graphical representations were performed in R 3.6.1 (R Foundation for Statistical Computing, Vienna, Austria). Significance was set at *p* < 0.05.

## 3 Results

### 3.1 Participant enrollment and descriptive statistics

Participants were excluded from the analysis if they: 1) did not have valid accelerometer data (*n* = 394), 2) had a clinical history of psychiatric disease (*n* = 28), 3) reported using sleeping pills (*n* = 7), 4) had missing data (*n* = 118), or 5) exhibited anomalous movement behaviors (e.g., average sleep duration < 1 h, SB or LPA = 0 min/day) (*n* = 5). The final analysis included 1095 workers.

The mean age was 50.2 ± 9.5 years, 68.6% were female, 23.4% were sales workers, and the majority of participants were college-educated, full-time employees (Table 1). Participants tended to sleep and engage in LPA for longer periods on non-workdays than on workdays, whereas SB and MVPA were lower on non-workdays.

**Table 1:** Characteristics of the study participants.

			*n* = 1095	
Mean age, years (SD)			50.2	(9.5)
Sex		Male	344	(31.4)
		Female	751	(68.6)
Mean (SD) body mass index, kg/m^2^			22.8	(3.7)
Mean (SD) education, years			14.7	(2.0)
Self-rated economic status		Good/Very good	766	(70.0)
		Poor/Very poor	329	(30.0)
Marital status		Married	740	(67.6)
		Unmarried	355	(32.4)
Smoking status		Never or former	936	(85.5)
		Current	159	(14.5)
Alcohol consumption		Never	210	(19.2)
		Sometimes	614	(56.1)
		Every day	271	(24.7)
Hiring status		Full-time employee	852	(76.3)
		Other	243	(23.7)
Job-type		Sales worker	256	(23.4)
		Other	839	(76.6)
Weekly overtime working hours		Not applicable (e.g., working as a freelancer)	65	(5.9)
		<10 h	752	(68.7)
		≥10 h	278	(25.4)
Psychological distress		Mean (SD) K6, points^a^	3.2	(3.4)
		Low (<5 points)	794	(72.5)
		High (≥5 points)	301	(27.5)
Work engagement (*n* = 1086)		Mean (SD) UWES, points	2.9	(1.4)
		Low (<3 points)	509	(46.9)
		High (≥3 points)	577	(53.1)
Mean (SD) accelerometer-wearing duration, days		Workday	10.6	(4.4)
		Non-workday	3.6	(2.2)
Mean (%)^b^ time-use composition, min/day				
	Workday	Sleep	344.4	(23.9)
		SB	699.8	(48.6)
		LPA	315.0	(21.9)
		MVPA	80.8	(5.6)
	Non-workday	Sleep	426.8	(29.6)
		SB	585.2	(40.6)
		LPA	362.2	(25.2)
		MVPA	65.8	(4.6)

SB, sedentary behavior; LPA, light-intensity physical activity; MVPA, moderate- to vigorous-intensity physical activity; K6, the 6-item Kessler Psychological Distress Scale; UWES, the Utrecht Work Engagement Scale.

Values are numbers (percentages) unless stated otherwise.

a A higher score indicates a higher level of psychological distress.

b Time-use composition presented as geometric means normalized to 24 h. The respective geometric means in percentages are presented in parentheses.

### 3.2 Associations between 24-hour movement behavior and mental health

Daily time-use composition was significantly associated with psychological distress and work engagement in both models on workdays only (Table 2, Table 3). In Model 2, more time spent sleeping was associated with low psychological distress (OR = 0.20, 95% CI = 0.10–0.44) and high work engagement (OR = 0.41, 95% CI = 0.20–0.81). By contrast, more time spent in SB was significantly associated with psychological distress (OR = 2.28, 95% CI = 1.23–4.28) and low work engagement (OR = 2.75, 95% CI = 1.55–4.91). More time spent in LPA was also associated with elevated psychological distress (OR = 2.45, 95% CI 1.46–4.17), but not with lower work engagement. Regarding time spent in MVPA, we found no significant association with positive or negative mental-health variables. Additionally, none of the behaviors were significantly correlated with mental health on non-workdays.

**Table 2:** Associations between 24-hour movement behaviors and psychological distress in workers (*n* = 1095).

		Model 1^a^					Model 2^b^				
		Odds ratio^d^	95% CI			*p*-value^e^	Odds ratio^d^	95% CI			*p*-value^e^
			Lower	–	Upper			Lower	–	Upper	
Behaviors in workday^c^											
	Sleep	**0.17**	**0.08**	**–**	**0.34**	**<0.001**	**0.20**	**0.10**	**–**	**0.44**	**<0.001**
	SB	**2.12**	**1.22**	**–**	**3.70**	**0.008**	**2.28**	**1.23**	**–**	**4.28**	**0.010**
	LPA	**2.73**	**1.84**	**–**	**4.09**	**<0.001**	**2.45**	**1.46**	**–**	**4.17**	**<0.001**
	MVPA	1.03	0.70	**–**	1.52	0.898	0.87	0.56	**–**	1.35	0.536
	Overall					**<0.001**					**<0.001**
Behaviors in non-workday^c^											
	Sleep	0.85	0.46	**–**	1.58	0.605	0.71	0.37	**–**	1.38	0.311
	SB	1.13	0.72	**–**	1.77	0.587	1.22	0.75	**–**	1.97	0.427
	LPA	1.31	0.87	**–**	1.98	0.205	1.37	0.87	**–**	2.17	0.181
	MVPA	**0.79**	**0.64**	**–**	**0.99**	**0.044**	0.84	0.66	**–**	1.08	0.170
	Overall					0.186					0.334

The results in bold are significant (*p* < 0.05).

a Model 1 included composition of 24-hour movement behaviors.

b Model 2 additionally included age, sex, body mass index, education years, self-rated economic status, marital status, health behaviors (alcohol consumption and smoking), job-type, hiring status, and average weekly overtime hours.

c Time-use composition was expressed as isometric log ratio (ilr) coordinates, and each result was from the initial ilr coordinates.

d The odds ratio corresponds to one unit increase in ilr coordinates. Low psychological distress was the reference category.

e *p*-values for individual ilr coordinates are from Wald tests, *p*-value for the overall composition is from the likelihood ratio test.

**Table 3:** Associations between 24-hour movement behaviors and work engagement in workers (*n* = 1086).

		Model 1^a^					Model 2^b^				
		Odds ratio^d^	95% CI			*p*-value^e^	Odds ratio^d^	95% CI			*p*-value^e^
			Lower	–	Upper			Lower	–	Upper	
Behaviors in workday^c^											
	Sleep	**0.33**	**0.17**	**–**	**0.62**	**<0.001**	**0.41**	**0.20**	**–**	**0.81**	**0.011**
	SB	**2.26**	**1.37**	**–**	**3.75**	**0.001**	**2.75**	**1.55**	**–**	**4.91**	**<0.001**
	LPA	1.13	0.80	**–**	1.60	0.478	0.86	0.54	**–**	1.37	0.524
	MVPA	1.19	0.84	**–**	1.69	0.332	1.03	0.70	**–**	1.53	0.864
	Overall					**0.002**					**0.005**

Behaviors in non-workday^c^											
	Sleep	0.87	0.50	**–**	1.51	0.611	0.68	0.38	**–**	1.22	0.197
	SB	1.35	0.91	**–**	2.03	0.142	1.36	0.88	**–**	2.11	0.165
	LPA	1.05	0.73	**–**	1.53	0.784	1.31	0.87	**–**	1.98	0.194
	MVPA	**0.81**	**0.66**	**–**	**0.99**	**0.043**	0.82	0.66	**–**	1.03	0.088
	Overall					0.063					0.128

The results in bold are significant (*p* < 0.05).

a Model 1 included composition of 24-hour movement behaviors.

b Model 2 additionally included age, sex, body mass index, education years, self-rated economic status, marital status, health behaviors (alcohol consumption and smoking), job-type, hiring status, and average weekly overtime hours.

c Time-use composition was expressed as isometric log ratio (ilr) coordinates, and each result was from the initial ilr coordinates.

d The odds ratio corresponds to one unit increase in ilr coordinates. High work engagement was the reference category.

e *p*-values for individual ilr coordinates are from Wald tests, *p*-value for the overall composition is from the likelihood ratio test.

**Fig. 1:**
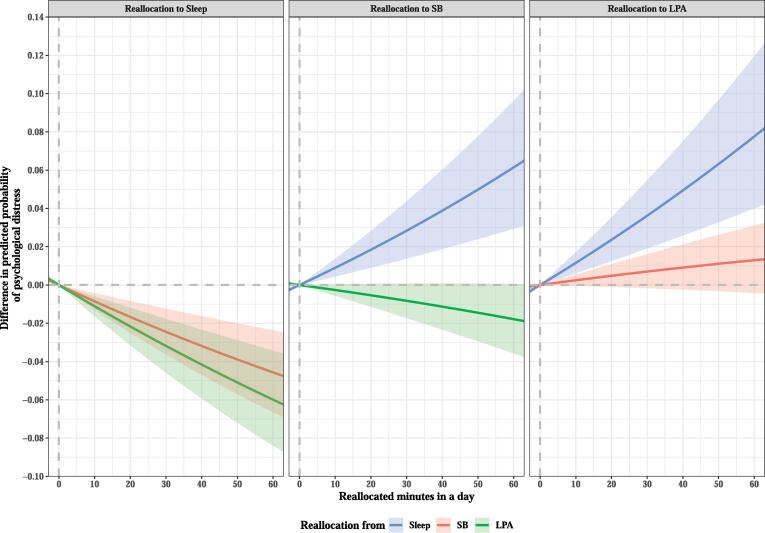
Difference in predicted probability of psychological distress when fixed amounts of time were reallocated between two movement behaviors in a workday while keeping the remaining components constant at compositional means. The analysis was based on Model 2. Only reallocation patterns that were found to be significantly associated with the first ilr-coordinate and psychological distress are shown in the graph. SB, sedentary behavior; LPA, light-intensity physical activity.

**Fig. 2:**
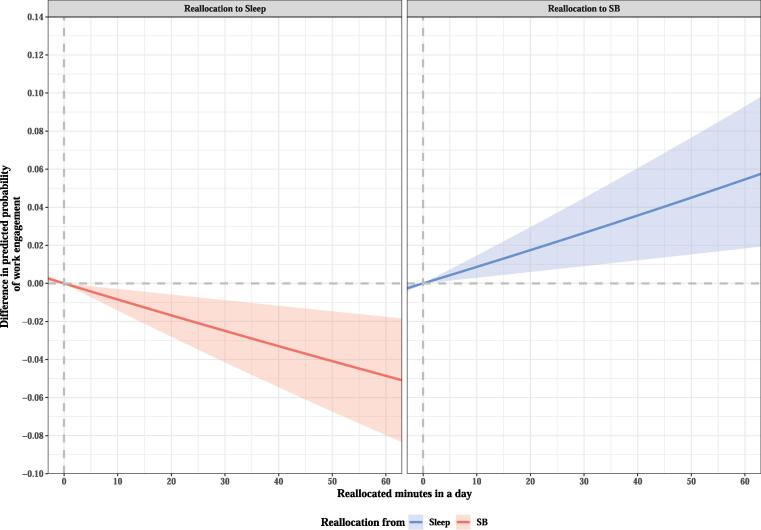
Difference in predicted probability of work engagement when fixed amounts of time were reallocated between two movement behaviors in workday while keeping the remaining components constant at compositional means. The analysis was based on Model 2. Only reallocation patterns that were found to be significantly associated with the first ilr-coordinate and work engagement are shown in the graph. SB, sedentary behavior.

Reallocation of time from SB and LPA to sleep was associated with decreased psychological distress (Fig. 1, Table A). Specifically, when 60 min/day of SB and LPA was reallocated to sleep, psychological distress was predicted to be 20.2% and 26.6% less likely, respectively. When 60 min/day of SB was reallocated to sleep, low work engagement was predicted to be 11.4% less likely (Fig. 2, Table B).

## 4 Discussion

This study is the first to examine the associations between 24-hour movement behaviors and positive and negative mental health in workers while accounting for the co-dependence of those behaviors in a given day using CoDA. We found new evidence that reallocating time from SB or LPA to sleep is associated with better mental health, reinforcing the importance of adequate sleep for managing mental health in workers.

We showed that time spent sleeping was associated with lower psychological distress and higher work engagement during workdays, whereas SB was linked to more stress and lower engagement. These results are consistent with well-documented findings using non-compositional analysis (Litwiller et al., 2017, Mullane et al., 2017, Munir et al., 2015, Zhai et al., 2015) and with a recent CoDA study on the general American adult population (del Pozo Cruz et al., 2020). Our data suggest that sleep and SB are important for managing mental health during workdays, but not during non-workdays.

We know little about the relationship between device-measured LPA and psychological distress. Only one non-compositional, cross-sectional study is available, showing that LPA is significantly associated with a low risk of psychological distress in adults (Hamer et al., 2014). However, two cross-sectional, compositional studies did not find a significant link between device-measured LPA and general mental health (McGregor et al., 2018) or depression symptoms (del Pozo Cruz et al., 2020). All three of these previous studies used samples from the general population through national representative surveys in developed countries, suggesting that participant characteristics are probably comparable. Therefore, inconsistencies between previous compositional and non-compositional studies might be due to differences in analytical procedures. In contrast, our CoDA findings likely differ from previous CoDA results (del Pozo Cruz et al., 2020, McGregor et al., 2018) because of differences in the study population. Participants in the earlier CoDA studies were sampled from multiple communities and held various jobs or were unemployed (e.g., homemakers and retired people). Here, however, we sampled workers with largely the same job in a single company. The unfavorable association of LPA with mental health in this study may be because LPA in a workplace reflects being busy, leading to greater work-related mental stress.

Although many epidemiological studies have suggested that engaging in MVPA (e.g., exercise) has beneficial effects on mental health (Mammen and Faulkner, 2013, Nishi et al., 2017), previous CoDA studies have reported inconsistent results. One study (del Pozo Cruz et al., 2020) found that more time spent in MVPA predicted lower depressive symptoms in adults, a result echoed in the elderly (McGregor et al., 2018). However, a separate study on Australian adults failed to identify such significant associations (Curtis et al., 2020). Notably, none of these previous reports focused specifically on workers. A previous meta-analysis suggested that the effects of physical activity on depression may be smaller in non-clinical populations than in clinical populations (Rebar et al., 2015). This study had a similar proportion of workers who met the criteria for poor mental health compared with the general Japanese population (Nishi et al., 2018, Shimazu et al., 2010), implying that participants’ psychological states were not very severe. We suggest that our sample characteristics and low statistical power may have partially contributed to the lack of any significant associations with MVPA.

Contrary to expectations, we did not observe any significant relationships between time use and mental health on non-workdays. Our results are partially consistent with the only non-compositional, cross-sectional study that examined the relationship between mental health and device-measured SB on non-workdays (Gibson et al., 2017). They also reported a nonsignificant relationship. We expect that time-use composition during non-workdays would vary widely between and within workers, compared with the relatively high consistency of workdays. Higher variability in how one spends time when not working may complicate capturing habitual time use and influence our results. Future studies should aim to investigate the effects of wider variation in leisure activities on the relationship between movement behaviors and mental health.

Our study indicates that a good strategy for managing mental health is to substitute time spent in SB or LPA with time spent sleeping. To facilitate this, employers could decrease their amount of overtime, thus lowering work-related SB and LPA. At home, workers could reduce SB, such as TV viewing and computer use, to increase the time available for sleep. Our results also suggest that obtaining sufficient time for sleep should be prioritized over MVPA when it comes to workplace mental-health management.

This study has several limitations. First, potential inaccuracies in behavioral data are may have occurred, given that self-reported sleep duration and device-measured daytime behaviors were scaled into a 24-hour period based on previously established CoDA methods (del Pozo Cruz et al., 2020, McGregor et al., 2019). Furthermore, because the hip-mounted accelerometer cannot fully distinguish between postural sitting and standing (Kurita et al., 2017), we could have misclassified SB and LPA occasionally. A second limitation is the cross-sectional nature of this study, which did not allow us to make causal inferences. To confirm the causality and validity of our findings, if possible, future studies using CoDA should be conducted with longitudinal data. A third limitation is that we excluded 34% of participants, increasing the possibility of sampling bias. Future studies may consider alternative strategies for dealing with missing data. Finally, our results may not be generalizable to all workers, because we used data from the employees of a single company who took an annual health check-up, all of whom lived in urban Japan.

In conclusion, we demonstrated that time allocation of 24-hour movement behaviors during workdays was significantly associated with mental health in workers. Specifically, time spent sleeping was favorably associated with mental health, whereas time spent in SB and LPA seemed to be detrimental. Our findings indicate that reallocating time from SB or LPA to sleep could decrease psychological distress while increasing work engagement, providing a potentially effective strategy for mental-health management in workers.

## CRediT authorship contribution statement

**Naruki Kitano:** Conceptualization, Methodology, Data curation, Formal analysis, Writing - original draft. **Yuko Kai:** Supervision, Investigation, Writing - review & editing. **Takashi Jindo:** Investigation, Writing - review & editing. **Kenji Tsunoda:** Writing - review & editing. **Takashi Arao:** Project administration, Writing - review & editing.

## Declaration of Competing Interest

The authors declare that they have no known competing financial interests or personal relationships that could have appeared to influence the work reported in this paper.

## Figures and Tables

**Table A:** Difference in predicted probability of psychological distress when fixed amounts of time between two movement behaviors in a workday, while keeping the remaining components constant at compositional means.

Changes (min/day)	To	Delta*	95% CI		To	Delta*	96% CI	
			Lower	Upper			Lower	Upper
Reallocation from Sleep…								
10	SB	0.0090	0.0044	0.0137	LPA	0.0117	0.0063	0.0173
20		0.0184	0.0091	0.0282		0.0228	0.0121	0.0341
30		0.0284	0.0139	0.0437		0.0344	0.0182	0.0517
40		0.0389	0.0188	0.0602		0.0505	0.0265	0.0768
50		0.0499	0.0240	0.0777		0.0633	0.0329	0.0967
60		0.0615	0.0294	0.0963		0.0765	0.0395	0.1177

Reallocation from SB…								
10	Sleep	−0.0086	−0.0129	−0.0043	LPA	0.0025	−0.0005	0.0056
20		−0.0167	−0.0250	−0.0084		0.0048	−0.0010	0.0108
30		−0.0245	−0.0363	−0.0125		0.0070	−0.0016	0.0159
40		−0.0319	−0.0470	−0.0163		0.0090	−0.0023	0.0209
50		−0.0390	−0.0571	−0.0201		0.0109	−0.0031	0.0257
60		−0.0457	−0.0665	−0.0237		0.0127	−0.0040	0.0305

Reallocation from LPA…								
10	Sleep	−0.0126	−0.0184	−0.0069	SB	−0.0024	−0.0052	0.0003
20		−0.0223	−0.0323	−0.0123		−0.0051	−0.0107	0.0006
30		−0.0317	−0.0455	−0.0176		−0.0078	−0.0164	0.0007
40		−0.0407	−0.0580	−0.0228		−0.0108	−0.0222	0.0008
50		−0.0522	−0.0736	−0.0296		−0.0139	−0.0282	0.0007
60		−0.0604	−0.0845	−0.0346		−0.0187	−0.0373	0.0004

This analysis was based on Model 2. CI, confidence interval; SB, sedentary behavior; LPA, light-intensity physical activity.

*Delta indicates the difference in predicted probabilities.

**Table B:** The difference in the predicted probability of work engagement when fixed amounts of time between two movement behaviors in a workday, while keeping the remaining components constant at compositional means.

Changes (min/day)	To	Delta*	95% CI	
			Lower	Upper
Reallocation from Sleep…				
10	SB	0.0086	0.0030	0.0145
20		0.0175	0.0060	0.0295
30		0.0265	0.0091	0.0448
40		0.0357	0.0122	0.0605
50		0.0451	0.0153	0.0766
60		0.0547	0.0185	0.0930

Reallocation from SB…				
10	Sleep	−0.0085	−0.0142	−0.0030
20		−0.0168	−0.0280	−0.0060
30		−0.0250	−0.0415	−0.0089
40		−0.0330	−0.0546	−0.0118
50		−0.0409	−0.0674	−0.0147
60		−0.0487	−0.0798	−0.0177

This analysis was based on Model 2. CI, confidence interval; SB, sedentary behavior.

*Delta indicates the difference in predicted probabilities.
